# Changes in the medical admissions and mortality amongst children in four South African hospitals following the COVID-19 pandemic: A five-year review

**DOI:** 10.1371/journal.pgph.0002829

**Published:** 2024-09-18

**Authors:** Kimesh Loganathan Naidoo, Jienchi Dorward, Kogielambal Chinniah, Melissa Lawler, Yugendhree Nattar, Christian Bottomley, Moherndran Archary

**Affiliations:** 1 Department of Paediatrics and Child Health, School of Clinical Medicine, College of Health Sciences, University of KwaZulu-Natal, Durban, South Africa; 2 King Edward VIII Hospital, Congella, Durban, South Africa; 3 Nuffield Department of Primary Care Health Sciences, University of Oxford, Oxford, United Kingdom; 4 Centre for the AIDS Programme of Research in South Africa (CAPRISA), Durban, South Africa; 5 Mahatma Gandhi Memorial Hospital, Phoenix, Durban, South Africa; 6 Prince Mshyeni Memorial Hospital, uMlazi, Durban, South Africa; 7 RK Khan Memorial Hospital, Chatsworth, Durban, South Africa; 8 London School of Hygiene & Tropical Medicine, London, United Kingdom; UNITED STATES OF AMERICA

## Abstract

Vulnerable children from poor communities with high HIV and Tuberculosis(TB) burdens were impacted by COVID-19 lockdowns. Concern was raised about the extent of this impact and anticipated post-pandemic surges in mortality. Interrupted time series segmented regression analyses were done using routinely collected facility-level data of children admitted for medical conditions at four South African referral hospitals. Monthly admission and mortality data over 60 months from 01 April 2018 to 31 January 2023 was analysed using models which included dummy lockdown level variables, a dummy post-COVID period variable, Fourier terms to account for seasonality, and excess mortality as a proxy for healthcare burden. Of the 45 015 admissions analysed, 1237(2·75%) demised with significant decreases in admissions during all the lockdown levels, with the most significant mean monthly decrease of 450(95%, CI = 657·3, -244·3) p<0·001 in level 5 (the most severe) lockdown. There was evidence of loss of seasonality on a six-month scale during the COVID periods for all admissions (p = 0·002), including under-one-year-olds (p = 0·034) and under-five-year-olds (p = 0·004). No decreases in mortality accompanied decreased admissions. Post-pandemic surges in admissions or mortality were not identified in children with acute gastroenteritis, acute pneumonia and severe acute malnutrition.During the COVID-19 pandemic, paediatric admissions in 4 hospitals serving communities with high levels of HIV, TB and poverty decreased, similar to global experiences; however, there was no change in in-hospital mortality. No post-pandemic surge in admissions or mortality was documented. Differences in the impact of pandemic control measures on the transmission of childhood infections and access to health care may account for differing outcomes seen in our setting compared to the global experiences. Further studies are needed to understand the impact of pandemic control measures on healthcare provision and transmission dynamics and to better inform future responses amongst vulnerable child populations.

## Background

The national lockdown regulations promulgated across the globe due to the COVID-19 pandemic disrupted essential healthcare services [1]. Emergency outpatient visits and admissions, decreased sharply among children in all countries, especially between February 2020 and December 2021 [2–5]. Decreases of 19%, 50% and 56% in paediatric admissions were documented in Cameroon, South Africa (SA) and across Europe, respectively, compared with pre-COVID-19 time periods [3–5]. Vulnerable populations, including children who have sub-optimal access to healthcare and who live in poverty, have higher rates of malnutrition and are seen in larger numbers in lower- and middle-income countries (LMICs). These sub-populations were especially negatively affected by the lockdowns[2–4].

The decrease in paediatric admissions has been greater in children with communicable (77%) compared with non-communicable diseases (37%) [5]. Children with lower respiratory tract infections (LRTI), including viral bronchiolitis, also decreased [6–8]. Changes in seasonal patterns of viral bronchiolitis when compared with patterns identified in previous pre-COVID-19 years were noted [6]. This was postulated to occur due to reduced person-to-person transmission, and it raised concerns that a rebound would occur when transmission mitigating strategies were curtailed [6].

Visits to children’s routine immunisation services, decreased significantly across multiple countries, after the start of the COVID-19 pandemic. The promulgation of national lockdown measures restricting movement and cancellation of public transport, at varying levels of severity occurred on 23 March 2020 [4,9]. These decreases were documented in both urban and rural primary healthcare facilities [10]. Outpatient visits for children with Human Immunodeficiency Virus (HIV) dropped by 41%, and antiretroviral treatment initiation of newly diagnosed children also decreased in 2020 and 2021 [11]. These changes in access and utilisation of preventative healthcare and HIV chronic care raised concerns for negative health consequences, especially where poverty, HIV and Tuberculosis (TB) are common and where many live in poverty in high-density communities [11]. HIV viral suppression rates, however, were shown to be maintained among children, suggesting some chronic disease programmes remained reasonably robust [12].

Overall, the COVID-19 pandemic disrupted healthcare provision and health-seeking behaviour and was postulated to disproportionately impact specific subpopulations in low-income countries with fragile health systems and pervasive social-structural vulnerabilities [13]. Documentation of these indirect effects of the COVID-19 pandemic has been largely restricted to the period during the peaks of the COVID-19 lockdowns between February 2020 and December 2021 and not adequately documented in communities with high burdens of HIV, Tuberculosis (TB) and malnutrition [11]. The impact of varying severities of national lockdowns on admissions and mortality is not known in vulnerable communities that rely on public transport. It is also not known whether the reduction in infectious diseases and a concomitant decrease in mortality due to an overall reduction of disease burden would occur in such communities Concern was also raised about the mortality and morbidity rates rising, specifically in these vulnerable children after the removal of lockdown measures [14].

Children hospitalised in specialist referral hospitals generally require higher levels of medical care and represent the more severe cases [15]. This study describes and analyses changes in admission and in-hospital mortality amongst children in South African specialist referral hospitals during the varying national lockdown levels of associated with the COVID-19 pandemic and the post-pandemic period and compares this with the pre-pandemic period.

## Methods

### Study design and population

We conducted an interrupted time series analysis of routinely collected facility-level data of children below the age of 13 years hospitalised across all four of the largest public sector (non-fee-paying) specialist referral hospitals in the city of Durban (eThekwini District), Kwa Zulu-Natal(KZN). The data included those hospitalised with medical diagnoses only, thus allowing analysis to reflect on the impact of the COVID-19 pandemic, specifically on communicable diseases. In-born neonates and children hospitalised for surgical (general surgery, trauma, ear nose and throat procedures, orthopaedic reasons) or other non-medical reasons (psychiatric and social admissions for respite care or neglect) were purposefully excluded from the analysis.

We used data from the King Edward VIII, Mahatma Gandhi Memorial, Prince Mshyeni Memorial and R K Khan Memorial hospitals, which provide 240 in-patient paediatric medical specialist care beds (including designated high care and beds for interim invasive ventilation) for approximately 1,1 million children [15,16]. The children admitted to these hospitals are referred by primary healthcare providers (nurse-run day clinics, family practitioners, non-specialist district hospitals) and are generally complex cases requiring higher care levels. Children who require longer-term invasive ventilation (>72 hours) are referred to paediatric intensive care units located at the quaternary hospital. The majority of the children who attend and are hospitalised in these four referral hospitals are from lower socio-economic communities and live in communities with high population densities [15]. A documented decline of 37% in routine immunisation coverage with a rapid recovery was seen in the Ethekwini district between April -June 2020 [4]. The HIV antenatal seroprevalence of the population served by these hospitals is high at 44·3%(CI;41·6–46·7), reflecting a high burden of both HIV-exposed infants and HIV-infected children [16,17].

The data for the period from 01 April 2018 to 31 January 2023 was retrospectively accessed from 07 February 2023 to 21 February 2023. The study period spanned 60 months and included 23 months in the pre-COVID-19 period (01 April 2018 to 28 February 2020), 23 months of the designated COVID-19 period (01 March 2020 to 31 January 2022), during which one of the five lockdown stages were promulgated and 14 months post COVID-19 period (01 February 2022 to 31 January 2023)when no lockdowns were in place [18,19]. Monthly data in the COVID period were thus stratified according to the predominant lockdown level in each of the 23 months in this period.

### Data collection

The admission diagnosis of children included in the facility-level monthly data was obtained from in-patient records that an attending paediatrician validated. Data on hospitalised children included children in all age groups below 13 years of age (SA’s referral hospitals have a 13-year-old cut-off for paediatric care), those below one year of age (infant) and those between one and five years of age. Data on hospitalised children under the age of five years with lower respiratory tract infections (LRTI) or acute gastroenteritis (AGE) as their main diagnosis were specifically tracked. In this study, the term LRTI as a diagnostic category includes patients with lobar or bronchopneumonia, bronchiolitis and bronchitis. This categorisation was based on a standardised nomenclature used by clinicians across all sampled hospitals in admission diagnoses and mortality classification. LRTI excludes upper respiratory tract infections (URTI) or upper airway obstruction, asthma or recurrent wheezing [20]. In addition, monthly admission numbers of children categorised as having severe acute malnutrition (SAM) using the WHO guidelines were also collected. In all four hospitals, the categorisation of a child under five years of age with SAM is verified by a paediatrician and then independently corroborated by an attending dietician within 72 hours post-admission. This dual verification for nutritional categorisation enables weights post-rehydration to be utilised and for lengths or heights to be rechecked for accuracy. In the WHO nutritional classification system, children are classified as either having severe acute malnutrition (SAM), moderate acute malnutrition (MAM), not acutely malnourished but considered at risk (NAM@risk), or not acutely malnourished (NAM) or as overweight or obese [21,22]. The SAM definition was based on weight-for-length z score and/or the presence of nutritional oedema as documented by an attending paediatrician [22]. The mid-upper arm circumference (MUAC) scores were not used in this study as the documentation was inconsistent in the reviewed source documents[21,22]. The numbers of children who demised monthly in all age categories and specifically those with a diagnosis of LRTI, AGE or SAM under the age of five years were also collected.

#### Verification of data

Four independent databases were utilised over the study periods [23]. These databases corroborated and validated information and ensured minimal missing data. Each hospital’s paediatric department has an in-hospital database used as the primary database. A specialist paediatrician in each hospital is responsible for verifying and entering all weekly admissions tallies and death information (categorised by age and diagnosis) from original case records into this primary database. Admission and mortality data is also verified monthly by paediatricians in the department from a standardised admission and deaths daily register and then submitted to a facility information officer, which feeds this data to a central district-wide district health information system database (DHIS) [23]. In this study, we validated the DHIS data obtained with source data in each hospital from the primary database that the attending paediatricians held to avoid inconsistencies. The third database was the Child Healthcare Problem Identification Programme (Child PIP). Paediatric departments across many SA hospitals utilise this database to record and systematically review child deaths independently, emphasising assessing modifiable factors related to these deaths [24]. Mortality figures per hospital were corroborated using the Child PIP and DHIS and verified at each hospital. The fourth database used verified nutritional categorisation of all in-hospital patients, and in-hospital dietitians maintained these databases in each hospital. The databases were rechecked and then verified with the hospital records for discrepancies.

### Data analysis and interpretation

We used descriptive statistics to summarise data and present summaries of admission, mortality and case fatality rates before, during and after the COVID-19 period with lockdowns. We did an interrupted time series segmented regression analysis by fitting linear regression models with the outcome of monthly paediatric admissions. The models included dummy lockdown level variables indicating 1 or 0 for each level 1 (least severe) to 5 (most severe) of lockdown and a dummy variable for the post-COVID-19 period. COVID-19 waves could also have caused an increased burden on the healthcare system, which may have affected paediatric healthcare use and admissions independently from lockdowns. We, therefore, modelled this by including a continuous variable for excess mortality in eThekwini for each month as a proxy for COVID-19-related burden on the healthcare system. To account for seasonal changes due to RSV and other respiratory virus outbreaks and Rotavirus and other viral causes of AGE, we included two pairs of sine and cosine terms (Fourier terms) in the models to account for seasonality. This approach takes account of pre-lockdown trends and allows estimation of the effect of each level of lockdown and whether there was a change in admissions during the period following the cessation of all lockdowns post-COVID. We built separate models by age (under one year, under five years and between 5 and 13 years) and diagnosis (LRTI, AGE and SAM). Age-specific changes thus do not sum to the total change because the total admissions (and deaths) were analysed as a separate time series. We checked for auto-correlation by calculating the auto-correlation and partial autocorrelation functions. We analysed data using R4.0 (R Foundation for Statistical Computing, Vienna, Austria).

### Ethical consideration

Adherence to ethical guidelines was ensured throughout the research process. The study was approved by the University of KwaZulu-Natal Biomedical Research Ethics Committee (BREC/00002981/2021), the KwaZulu-Natal Department of Health’s Provincial Health Research Ethics Committee, eThekwini District Health Department and the Child Health Identification Programme (National committee) with a waiver for informed consent for analysis of anonymised, routinely collected data.

## Results

During the 60-month study period that extended from 01 April 2018 to 31 March 2023, 45 015 children were admitted across all four specialist hospitals in Durban (eThekwini district). Of these, 20·490 (45·5%) were <1 year of age(infants), 16 549 (36.8%) were children between one and below five years, and 7976(17·7%) were children between five and below 13 years. Across all these age groups, 1237 children died in hospital during the 60 months of the study period, with 733(59·3%) being infants, 346(28%) between one and below five years and 158(12·7%) between five and 13 years. Table 1 compares unadjusted mean monthly admission and mortality numbers and Table 2 compares raw case fatality rates during the three assessed periods. While the mean monthly admission appeared marginally lower in the COVID-19 period, there was less of a decrease in mean monthly deaths. The case fatality rates for LRTI, AGE and SAM in the under-five-year group were higher during COVID-19.

**Table 1:** Unadjusted mean monthly admission and mortality numbers during all lockdown levels and post-COVID period.

Mean Monthly numbers	Pre-COVID	COVID	Post -COVID
	01 Apr 2018 to 28 Feb 2020	01 Mar 2020 to 31 Jan 2022	01 Feb 2022 to 31 Jan 2023
Admissions—children less than 13 years of age	871.3	589.8	814.8
Admissions—children between 5 and 13-years of age	155	101.3	148.7
Admissions—children below the age of 5 years	716.3	488.5	666.1
Admissions below the age of one year	382.9	274.3	383.9
Lower-respiratory tract infections admissions in 1-5-years olds	194.9	131.3	209.4
Acute Gastroenteritis admissions in 1-5-years olds	154.7	102.1	139.9
Severe acute malnutrition admissions in 1-5-years olds	42.7	28.1	40.2
Deaths—children less than 13 years of age	22.0	19.1	20.8
Deaths—children below the age of 5-years of age	19	16,6	18,6
Deaths—children less than 1 year of age	12.4	10.9	14.1

**Table 2:** Case fatality rates of under 5 year old children across the pre COVID-19, COVID-19 lockdown and post COVID periods.

Case Fatality Rates in percentages of under 5 year old children for Acute Gastroenetritis, Lower Respiratory Tract infections and Severe acute Malnutrition admissions			
Periods	Pre-COVID	COVID	Post -COVID
Dates	01 Apr 2018 to 28 Feb 2020 (%)	01 Mar 2020 to 31 Jan 2022 (%)	01 Feb 2022 to 31 Jan 2023 (%)
Acute Gastroenteritis (case fatality rates in under-5- year olds)	2.0	2.8	2.3
Lower-respiratory tract infections (case fatality rates in under-5- year in olds)	1.7	2.7	2.1
Severe acute malnutrition (case fatality rates in under-5- year -olds)	9.5	12.1	10.5

### Interrupted time series analysis

#### Adjusted admission and mortality monthly numbers by age group

**Fig 1:**
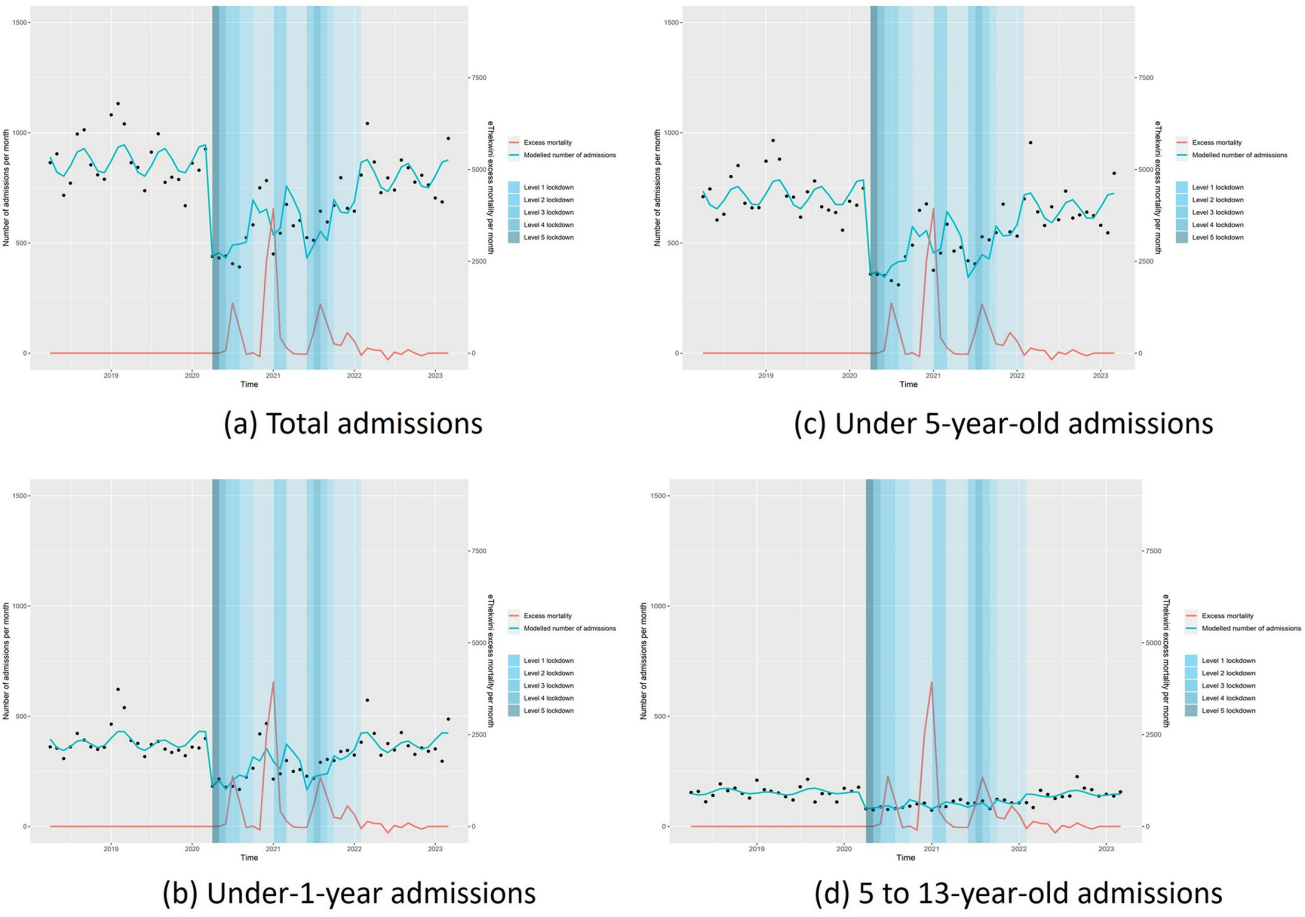
Interrupted time series analysis of admissions by age group, all ages (a), under 12 months (b), under 60 months (c) and between 5 and 13 years old (1d).

The analysis showed a significant decrease in total admissions for children under 1, 1-to-5-year-olds, and 5-13-year-olds. Level 5 lockdowns saw the most significant mean monthly decreases of 450(95% CI = -657·3–-244·3) p<0·001, 213.2(-349–-76·8) p = 0·003, 376·4(- 566·3–-186·4) p<0·0001 in total, under-1-year-old and 1-to-5-year-old admissions respectively. Level-1-lockdowns had the lowest mean monthly decreases. The trend was similar for school-going children (5-13-year olds) to all other age groups. There was also evidence of seasonality on a 6-month scale during the pre-and post-COVID periods in total admissions (p = 0·002), under-1-year-olds (p = 0·034) and 1-to-5-year olds (p = 0·004) which was not evident during the COVID-19 lockdown periods. In the segmented regression model, there was no evidence that excess monthly mortality in SA was associated with changes in admission numbers in any age group. Fig 1A–1D illustrates these findings.

In the post-COVID-19 period, total admissions remained slightly lower than during the pre-COVID-19 period (decrease of 68, 95% CI = -134·2–-2·4, p = 0·0430). This was mainly due to a decrease in the 1-5-age group (-60·9, 95% CI = -121·5–0·2, p = 0.049), with no evidence of a difference in the under-1-age group (+6.5, -50·1–37·0, p = 0·765), nor 5-13-year olds (-9·6, - 26·8–7.7, p = 0·270).

**Fig 2:**
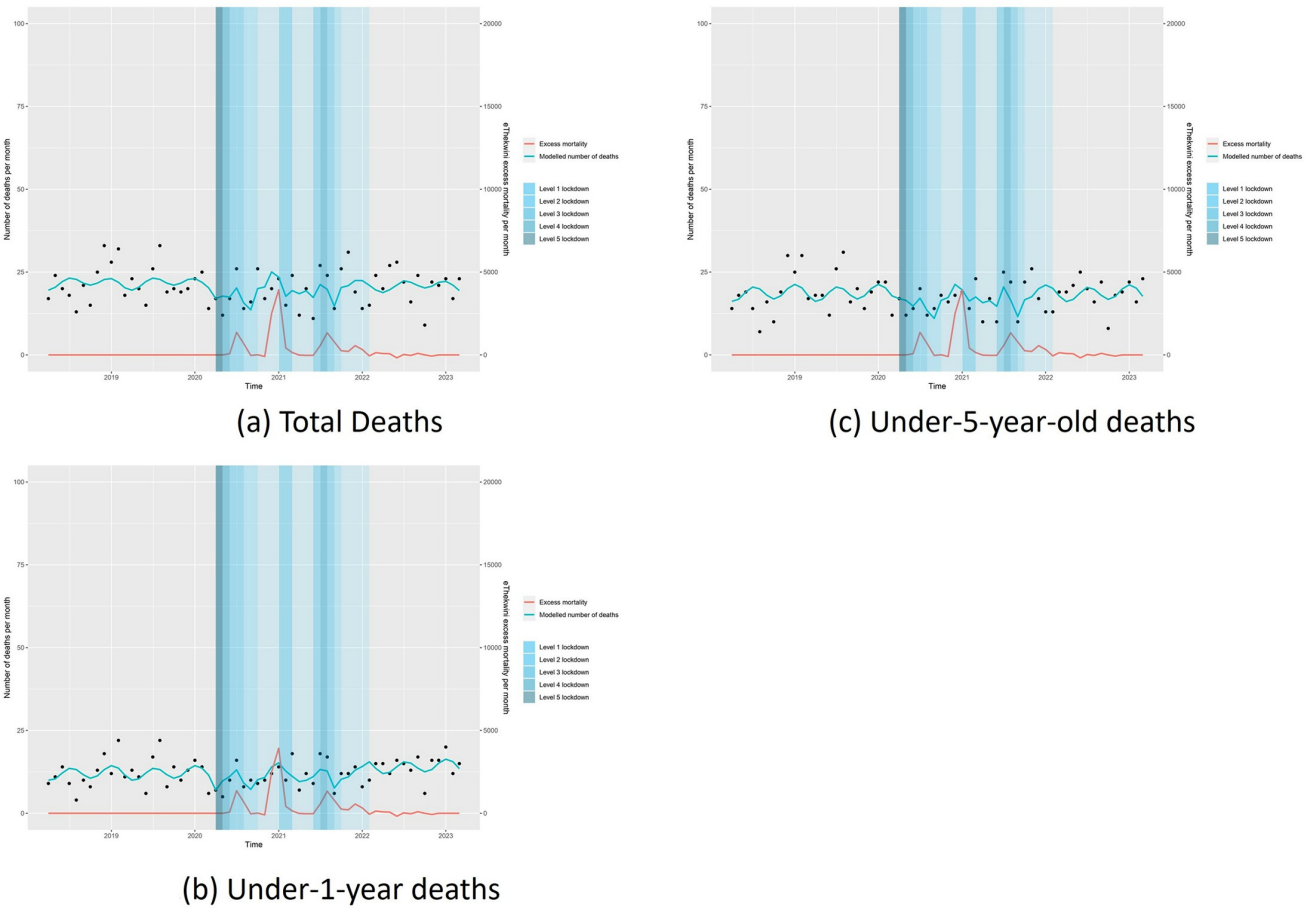
Interrupted time series analysis of mortality rates by age group all ages (a), under 12 months (b) and 60 months (c).

The segmented regression analysis showed no significant change in monthly mortality in all ages nor specifically in the age categories of under-1-year-olds and 1-to-5-year-olds and 5-13-year age groups during any lockdown levels, nor the post-COVID period. (Table 3, Fig 2A–2C provide the data and illustrate the trends, respectively).

**Table 3:** Comparison of mean monthly admissions and mortality in lockdown levels 1 to 5 and the post-COVID period with pre COVID-19 period.

Comparison of mean monthly admissions and mortality in lockdown levels 1 to 5 and the post-COVID period with pre COVID-19 period						
	Total (all below 13 years)		Under-1-year		1–5 year	
	Admissions	Mortality	Admissions	Mortality	Admissions	Mortality
Level 1	-188.1 CI -267.8, -108.4 p<0.001	-1.0 CI -5.7, 3.7 p = 0.657	-58.9 CI -111.5, -6.3 p = 0.029	-0.4 CI-3.7, 2.9 p = 0.795	-144.7 CI-218.0, -71.4 p<0.001	-0.4 CI -4.8, -4.0 p = 0.852
Level 2	-423.8 CI -556.9, -290.7 p<0.001	-8.0 CI-15.9, -0.2 p = 0.045	-167.2 CI-255.2, -79.3 p<0.001	-4.4 CI -9.9, 1.1 p = 0.114	-336.3 CI -458.8, -213.9 p<0.001	-7.0 CI -14, -0.4 p = 0.064
Level 3	-371.7 CI -482.2, -261.1 p<0.001	-4.8 CI -11.3, 1.7 p = 0.146	-178.3 CI -251.3, -105.2 p<0.001	-1.1 CI -5.7, 3.4 p = 0.628	-311.1 CI-412.8, -209.4 p<0.001	-4.3 CI -10.4, -1.9 p = 0.167
Level 4	-365.9 CI-516.1, -215.7 p<0.001	-2.7 CI -11.5, 6.2 p = 0.546	-148.8 CI-248.1, -49.6 p = 0.004	-0.6 CI -6.8, 5.6 p = 0.845	-304.7 CI-442.9, -166.5 p<0.001	-0.3 CI -8.7, -8.0 p = 0.935
Level 5	-450.8 CI 657.3, -244.3 p<0.001	-2.5 CI -14.7, 9.6 p = 0.677	-213.2 CI-349.6, -76.8 p = 0.003	-3.0 CI-11.5, 5.5 p = 0.483	-376.4 CI-566.3, -186.4 p<0.001	0.8 CI -10.6, -12.3 p = 0.885
Post-COVID period	-68.3 CI-134.2, -2.4 p = 0.043	-0.8 CI -4.7, 3.0 p = 0.665	6.5 CI -50.1, 37.0 p = 0.765	2.0 CI -0.8, 4.7 p = 0.155	-60.9 CI -121.5, -0.2 p = 0.049	-0.1 CI -3.8, -3.5 p = 0.950

*p<0.05 = significant.

#### Admission and mortality rates for children with acute gastroenteritis (AGE), lower respiratory tract infections (LRTI)and severe acute malnutrition (SAM)

Significant decreases in admissions were seen during most of the lockdown levels in the COVID-19 period in children hospitalised with AGE, LRTI or SAM (Table 4). Level 5 lockdowns saw decreases of 82·8(95%, CI = 156·3–-9·3) p = 0·028, 132·8(-238·6–-27·0)p = 0·015 and 25·7(95%, -47·4–-3·9), p = 0·022 in AGE, LRTI and SAM cases. The terms for seasonality provided evidence of seasonal variation on both a 6-month (p = 0·032) and 12-month (p = 0·003) scale for AGE admissions, a 6-month scale for LRTI admissions (p = 0·004) and a 12-month scale for SAM admissions (p<0·001).

**Table 4:** Comparison of adjusted Mean Monthly Admission and Case Mortality Numbers in 1–5 years children during all lockdown levels and the post-COVID -period with the pre COVID period.

Comparison of adjusted Mean Monthly Admission and Case Mortality Numbers in 1–5 years children during all lockdown levels and the post-COVID -period with the pre COVID period						
	Acute Gastroenteritis		Lower Respiratory tract infections		Severe acute Malnutrition	
	Admissions	Mortality	Admissions	Mortality	Admissions	Mortality
Level 1	-26.0 CI-54.4, 2.4 p = 0.071	0.3 CI-1.4, 1.9 p = 0.764	-47.7 CI -88.5, -6.9 p = 0.023	0.8 CI -1.1, 2.6 p = 0.428	-9.7 CI-18.1, -1.3 p = 0.024	-0.1 CI-1.8, 1.6 p = 0.906
Level 2	-123.7 CI -171.1, -76.3 p<0.001	-1.1 CI -3.9, 1.8 p = 0.453	-89.3 CI-157.5, -21.1 p = 0.011	-3.1 CI-6.3, 0.0 p = 0.051	-15.6 CI-29.6, -1.6 p = 0.030	0.7 CI-2.2, 3.5 p = 0.632
Level 3	-107.0 CI -146.3, -67.6 p<0.001	-2.2 CI -4.6, 0.1 p = 0.065	-121.1 CI-177.7, -64.5 p<0.001	0.7 CI-2.0, 3.3 p = 0.618	-19.2 CI-30.9, -7.6 p = 0.002	-0.4 CI-2.8, 1.9 p = 0.726
Level 4	-52.7 CI-106.1, 0.8 p = 0.053	1.5 CI-1.6, 4.7 p = 0.335	-99.8 CI-176.8, -22.8 p = 0.012	0.0 CI -3.6, 3.5 p-0.994	-14.0 CI -29.8, 1.8 p = 0.082	0.8 CI -2.4, 4.0 p = 0.617
Level 5	-82.8 CI -156.3, -9.3 p = 0.028	-1.4 CI-5.8, 3.0 p = 0.527	-132.8 CI -238.6,-27.0 p = 0.015	-2.9 CI-7.8, 2.0 p = 0.244	-25.7 CI -47.4, -3.9 p = 0.022	2.7 CI-1.7, 7.1 p = 0.224
Post-COVID period	-19.3 CI -42.7, 4.2 p = 0.105	-0.2 CI-1.6, 1.2 p = 0.808	9.0 CI -24.7, 42.8 p = 0.593	1.2 CI -0.3, 2.8 p = 0.123	-4.3 CI -11.3, 2.6 p = 0.216	0.1 CI -1.3, 1.5 p = 0.898

*p<0.05 = significant.

**Fig 3:**
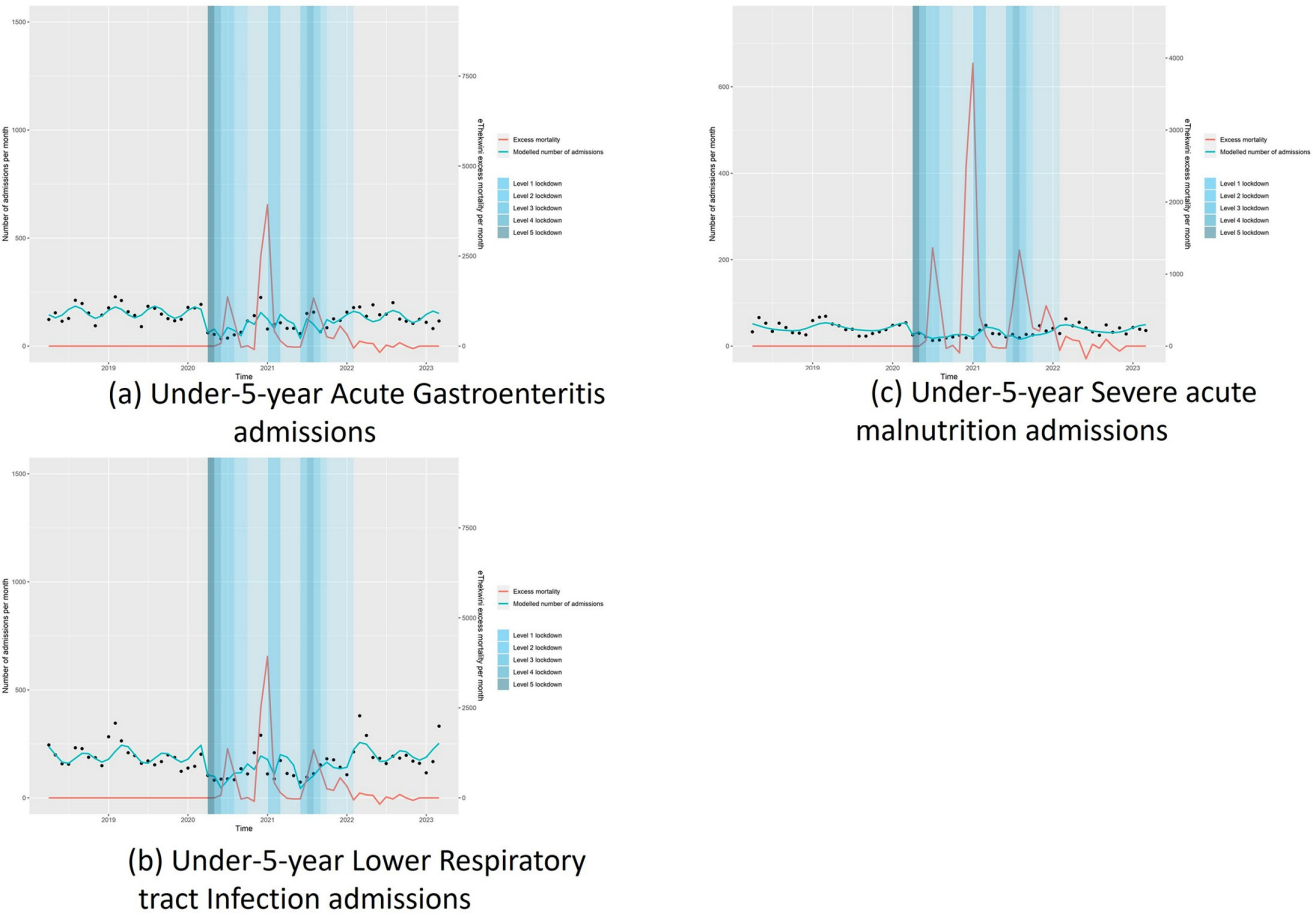
Interrupted time series analysis of admissions by diagnosis in 1–5 year old with acute gastroenteritis (a), lower respiratory tract infections (b) and severe acute malnutrition (c).

Fig 3A–3C illustrate these changes and loss of the seasonal patterns in AGE and LRTI seen during the COVID-19 period.

In the post-COVID period, there was no evidence that AGE, LRTI, or SAM admissions changed compared to pre-COVID numbers. Fig 3A–3C illustrate a return to seasonal patterns in the post-COVID period for cases of AGE and LRTI.

**Fig 4:**
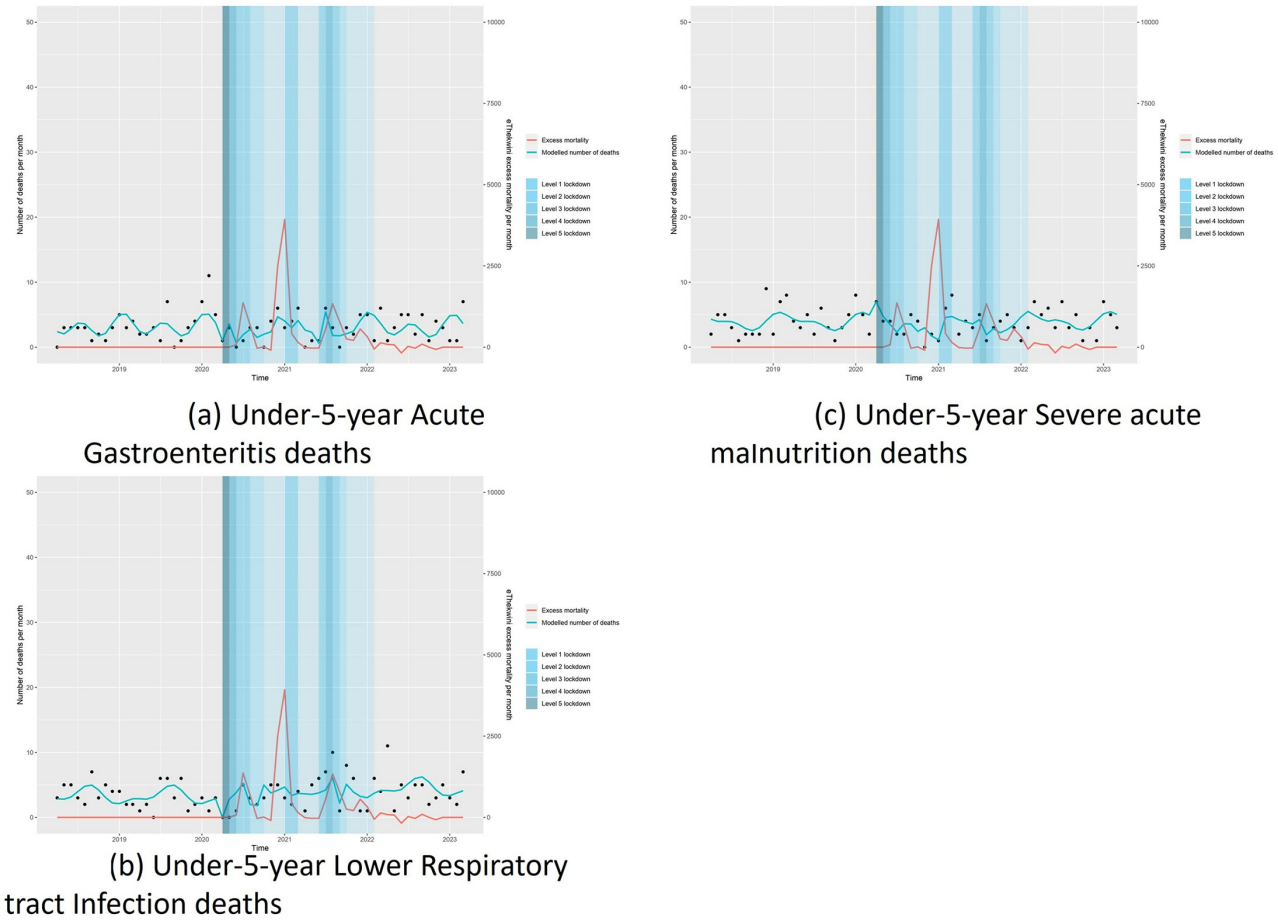
Interrupted time series analysis of mortality rates per admission diagnosis in 1–5 year-olds with acute gastroenteritis (a), lower respiratory tract infections (b) and severe acute malnutrition (c).

When analysing changes in mortality in those hospitalised with either AGE, LRTI or SAM, no significant changes were noted in all the lockdown levels. Table 4 and Fig 4A–4C illustrates these findings.

## Discussion

Our analysis shows that changes in patterns of admissions and mortality of vulnerable SA children following the COVID-19 pandemic do differ from experiences elsewhere in the world. Despite significant decreases in admissions and changes in seasonal patterns of communicable diseases during the COVID-19 lockdowns, there was neither a concomitant decrease in in-hospital deaths nor was there an anticipated post-pandemic surge in admissions in children from communities with high levels of HIV, TB and poverty.

Several modelling studies and early reviews from LMICs have raised concerns about the impact of the COVID-19 pandemic on vulnerable populations, especially those where fragile healthcare systems exacerbate delayed access to care [10,25]. In our study reflecting sick children requiring hospital admission and drawn from low-income communities, a high population density and existing infectious burden admission numbers did decrease, as was documented in high-income countries, following the promulgation of stringent lockdowns [5,13,15,26]. These decreases in admissions at referral hospitals mirrored decreases in admissions and visits to primary health clinics [4]. Of concern, however, is that the documented decrease in primary care visits and referral hospital admissions could reflect decreased access to healthcare for sick children. Whilst lockdown laws permitted the seeking of healthcare and all facilities remained open through the COVID-19 pandemic, the significant decrease in the admission of sick children raises the likelihood of worsening access to healthcare amongst vulnerable populations. In addition to concerns about decreased access to health care, these findings may reflect the influence of a decreased transmission of common childhood communicable diseases, possibly affected by decreased social interactions and mitigating strategies to prevent COVID-19 transmission [9,10]. It has been postulated that increased preventative hygiene habits adopted during the COVID-19 period, like masking, regular hand washing, creche and school closures, and other restrictions impacting person-to-person spread of infections, resulted in modified seasonal patterns of communicable diseases like Rotavirus associated AGE and Respiratory syncytial Virus associated LRTI [6,8,27]. The impact of this possible outcome, however, has not been fully understood in vulnerable child populations, including those with high population densities.

In this study, which reflects children admitted at referral hospitals, including those with complex problems and diagnoses, mortality numbers in all age groups and children with AGE, LRTI and SAM did not decrease during the lockdown period, unlike previously reported [10]. Our finding of the persistence of high mortality despite significant decreases in admissions in the COVID-19 period has been documented elsewhere in poor socio-economic communities [3]. The concern with this finding is that children who became sick presented later and were more unwell and were thus more likely to die. Concerns that increases in child mortality may have been seen out of hospitals and in intensive care units are not borne out however by any significant increase in excess childhood mortality as seen in age-specific annualised excess death rates (per 1000 population) documented over this period from both the community and hospitals [28].

We postulate that in our large cohort of children hospitalised in public sector referral hospitals, there are many children, especially those living within high population densities, who continued to have exposure to many childhood infections and continued to have delayed access to care for a multiplicity of reasons. This latter group has been previously documented as experiencing delays in accessing standard healthcare despite the availability of free public health services [29]. Many caregivers here are noted to utilise multiple other sources of care, including allopathic, indigenous and home treatments, before presenting at public services, often with severe complications or in severe distress [29]. It is possible that caregivers in this sub-group would have persisted with late presentation for acute care, similar to pre-pandemic behaviours or delayed their access to hospital care even later. Further exploration is thus required to determine how this vulnerable group were uniquely affected by the challenges posed both by the COVID-19 pandemic and the associated lockdowns.

Our study also documents that the expected surge in malnutrition cases during the lockdown period did not occur, unlike those reported in other studies from developing countries [10,30]. The unadjusted higher case fatality rates in SAM in the COVID-19 period cases despite decreased admissions however suggests that those malnutrition cases that were admitted were likely more severe, presenting later in their disease course and hence had a higher likelihood of death, corroborating the findings where vulnerable children were severely affected by the lockdown measures [28].

Decreased utilisation of routine immunisation services with a subsequent post-pandemic surge in vaccine-preventable infections was a major global concern [11,25,31,32]. With the disruption of seasonal patterns of viral bronchiolitis, an expected surge in LRTI was also anticipated and documented across countries with severe lockdowns [33,34]. In this study, which analysed data over a longer post-pandemic period than most other studies, we do not show this anticipated surge in admissions in under-1-year and 1-to-5-year-old children as well as in cases of AGE, LRTI and SAM. The trend identified in the post-pandemic period may reflect a gradual increase in admissions back to pre-COVID levels. We postulate that these vulnerable children living in high population densities with cramped living were exposed to common childhood infections, unlike those children living in low population densities, and the lack of a post-pandemic surge seen with most of the children in this cohort could be explained by this possibility. Additionally, the rapid initiation of a catch-up immunisation program to return immunisation coverage to prepandemic levels in KZN may have played a role in the absence of a post-pandemic surge in infections seen in this study. [4,31,32].

Strengths of our study include the large cohort of children hospitalised specifically for medical diagnoses in public sector referral hospitals. We reflected on acutely sick and vulnerable children susceptible to communicable diseases. Our use of long-term routine data considers pre-COVID, all the COVID lockdown levels, and substantial post-COVID periods, whilst most studies have largely focused on the COVID-19 period. However, we were not able to verify definitive microbiological, virologic and formal HIV and TB results; instead, we relied on retrospective diagnoses provided by source documents and by paediatricians on site. Further studies specifically targeting these populations with verifiable microbiological testing may be required to unpack children’s behaviours under differing contexts. We further extrapolated immunisation coverage of the study population on district-wide data. This study may help determine the epidemiological patterns of vulnerable children when faced with communicable disease outbreaks in greater detail. We did not focus on neonatal or non-medical admissions or children admitted to intensive care units (ICU) requiring ventilation. Access to intensive care units in our resource-poor areas is limited with only 25 paediatric intensive care beds in KZN, (0.73 beds ICU per 100 000 children), thus our data does reflect the majority of sick admissions [35]. We could not assess the definitive socio-economic status and inferred this based on previous usage patterns in public sector hospitals. The retrospective data reflects in-hospital mortality specifically and does not include community-based death data.

In conclusion, our findings suggest that, in one of the regions most affected by HIV, Tuberculosis and malnutrition, whilst admissions of acutely sick children decreased similar to other countries with better health resources, a decrease in in-hospital mortality and anticipated post-pandemic surges in admission was not seen as compared with these countries. This study provides evidence that children in vulnerable communities with high population densities of HIV and TB infection rates behaved differently in communities where these conditions were not as common. These findings suggest that mitigating strategies to reduce infectious disease outbreaks possibly affected transmission dynamics of common communicable childhood diseases differently in communities, and this requires further exploration and study. Further studies in vulnerable populations are needed to identify persisting challenges in healthcare provision, infection transmission dynamics and the impact of promulgation of uniform pandemic control measures on child health outcomes.

## Supporting information

S1 File (XLSX)

## References

[1] Haider N, Osman AY, Gadzekpo A, Akipede GO, Asogun D, Ansumana R, et al. Lockdown measures in response to COVID-19 in nine sub-Saharan African countries. BMJ Global Health 2020; 5: e003319. doi: 10.1136/bmjgh-2020-003319 33028699 PMC7542624

[2] Jensen C, Yannigan Y, Mafanya N, Majozi N, Martin T, Mnguni T, et al. Patterns of disease on admission to children’s wards and changes during a COVID-19 outbreak in KwaZulu-Natal Province, South Africa. S Afr Med J. 2022;112(4):279–287. 35587807

[3] Chiabi A, Forgwei M, Bissong M, Niba L, Abah J, Nsame D. Trends in Pediatric Admissions and Mortality during the COVID-19 Pandemic in an Urban Setting in Cameroon. J Trop Pediatr. 2022; 68(3):fmac026. doi: 10.1093/tropej/fmac026 35348796 PMC8992244

[4] Jensen C., & McKerrow N. (2020). Child health services during a COVID-19 outbreak in KwaZulu-Natal Province, South Africa. S Afr Med J, 111(2), 114–119. doi: 10.7196/SAMJ.2021.v111i2.15243 http://www.samj.org.za/index.php/samj/article/view/13185. 33944720

[5] Kruizinga M, Peeters D, van Veen M, van Houten M, Wieringa J, Noordzij J, et al. The impact of lockdown on pediatric ED visits and hospital admissions during the COVID-19 pandemic: a multicenter analysis and review of the literature. Eur J Pediatr. 2021; 180(7):2271–2279. doi: 10.1007/s00431-021-04015-0 33723971 PMC7959585

[6] Van Brusselen D, De Troeyer K, Ter Haar E, Vander Auwera A, Poschet K, Van Nuijs S, et al. Bronchiolitis in COVID-19 times: a nearly absent disease? Eur J Pediatr. 2021;180(6):1969–1973. doi: 10.1007/s00431-021-03968-6 33517482 PMC7847293

[7] Coma E, Méndez-Boo L, Mora N, Guiriguet C, Benítez M, Fina F, et al. Divergences on expected pneumonia cases during the COVID-19 epidemic in Catalonia: a time-series analysis of primary care electronic health records covering about 6 million people. BMC Infect Dis. 2021;21(1):283. doi: 10.1186/s12879-021-05985-0 33740907 PMC7979451

[8] Curatola A, Lazzareschi I, Bersani G, Covino M, Gatto A, Chiaretti A. Impact of COVID-19 outbreak in acute bronchiolitis: Lesson from a tertiary Italian Emergency Department. Pediatr Pulmonol. 2021;56(8):2484–2488. doi: 10.1002/ppul.25442 33961732 PMC8242382

[9] Dorward J, Khubone T, Gate K, Ngobese H, Sookrajh Y, Mkhize S, et al. The impact of the COVID-19 lockdown on HIV care in 65 South African primary care clinics: an interrupted time series analysis. Lancet HIV. 2021;8(3):e15–-e165. doi: 10.1016/S2352-3018(20)30359-3 33549166 PMC8011055

[10] McIntosh A, Bachmann M, Siedner MJ, Gareta D, Seeley J, Herbst K. Effect of COVID-19 lockdown on hospital admissions and mortality in rural KwaZulu-Natal, South Africa: interrupted time series analysis. BMJ Open. 2021;11(3):e047961. doi: 10.1136/bmjopen-2020-047961 33737445 PMC7977076

[11] Nachega JB, Kapata N, Sam-Agudu NA, Decloedt EH, Katoto PDMC, Nagu T, et al. Minimising the impact of the triple burden of COVID-19, tuberculosis and HIV on health services in sub-Saharan Africa. Int J Infect Dis. 2021;113(Suppl 1):S16–S21. doi: 10.1016/j.ijid.2021.03.038 33757874 PMC7980520

[12] Mathamo A, Naidoo KL, Dorward J, Archary T, Bottomley C, Archary M. COVID-19 and HIV viral load suppression in children and adolescents in Durban, South Africa. South Afr J HIV Med. 2022;23(1):1424. doi: 10.4102/sajhivmed.v23i1.1424 36575700 PMC9772656

[13] Govender K, Cowden RG, Nyamaruze P, Armstrong RM, Hatane L. Beyond the Disease: Contextualised Implications of the COVID-19 Pandemic for Children and Young People Living in Eastern and Southern Africa. Front Public Health. 2020;8:504. doi: 10.3389/fpubh.2020.00504 33194933 PMC7604346

[14] Nachega JB, Grimwood A, Mahomed H, Fatti G, Preiser W, Kallay O, et al. From Easing Lockdowns to Scaling Up Community-based Coronavirus Disease 2019 Screening, Testing, and Contact Tracing in Africa-Shared Approaches, Innovations, and Challenges to Minimise Morbidity and Mortality. Clin Infect Dis. 2021;72(2):327–331. doi: 10.1093/cid/ciaa695 33501963 PMC7314180

[15] Massyn N, Barron P, Day C, Ndlovu N, Padarath A, editors. District Health Barometer 2018/19. Durban: Health Systems Trust, February 2020. https://www.hst.org.za/publications/District%20Health%20Barometers/District+Health+Barometer+2018-19+Web.pdf.

[16] Hoque M, Hoque ME, van Hal G, Buckus S. Prevalence, incidence and seroconversion of HIV and Syphilis infections among pregnant women of South Africa. S Afr J Infect Dis. 2021;36(1):296. doi: 10.4102/sajid.v36i1.296 34917677 PMC8661397

[17] Human Sciences Research Council. South African National HIV Prevalence, Incidence, Behaviour and Communication Survey, 2017. http://www.hsrc.ac.za/uploads/pageContent/10779/SABSSM%20V.pdf.

[18] Republic of South Africa. Government Gazette Vol. 451 Cape Town, 15 January 2003, No. 24252. No. 57 of 2002: Disaster Management Act, 2002. https://www.cogta.gov.za/cgta_2016/wp-content/uploads/2016/06/DISASTER-MANAGEMENT-ACT.pdf.

[19] Republic of South Africa. COVID-19 / Novel Coronavirus—Regulations and Guidelines—Coronavirus COVID-19. https://www.gov.za/covid-19/resources/regulations-and-guidelines-coronavirus-covid-19.

[20] Bamford LJ, Barron P, Kauchali S, Dlamini NR. In-patient case fatality rates improvements in children under five: diarrhoeal disease, pneumonia and severe acute malnutrition. S Afr Med J. 2018;108 (3 Suppl 1):S33–S37. doi: 10.7196/SAMJ.2018.v108i3.12772

[21] World Health Organization (WHO)/United Nations Children’s Fund (UNICEF). WHO Growth Standards and the Identifcation of Severe Acute Malnutrition in Infants and Children, 2009. https://iris.who.int/bitstream/handle/10665/44129/9789241598163_eng.pdf?sequence=1. 24809116

[22] KZN Department of Health. KZN Guidelines on the Integrated Management of Acute Malnutrition (IMAM), January 2014. http://www.kznhealth.gov.za/family/mcwh/kzn-imam-guidelines.pdf.

[23] Bamford LJ, McKerrow NH, Barron P, Aung Y. Child mortality in South Africa: Fewer deaths, but better data are needed. S Afr Med J. 2018;108(3 Suppl 1):S25–S32. doi: 10.7196/SAMJ.2017.v108i3b.12779

[24] Stephen CR. Saving Children 2012–2013: An Eighth Survey of Child Healthcare in South Africa. Pretoria: Tshepesa Press, 2016. https://www.up.ac.za/media/shared/717/Child%20PIP/Saving%20children%20reports/saving-children-2012-2013.zp207156.pdf.

[25] Roberton T, Carter ED, Chou VB, Stegmuller AR, Jackson BD, Tam Y, Sawadogo-Lewis T, Walker N. Early estimates of the indirect effects of the COVID-19 pandemic on maternal and child mortality in low-income and middle-income countries: a modelling study. Lancet Glob Health. 2020 Jul;8(7):e901–e908. Epub 2020 May 12. doi: 10.1016/S2214-109X(20)30229-1 . 32405459 PMC7217645

[26] Hu N, Nassar N, Shrapnel J, Perkes I, Hodgins M, O’Leary F, et al. The impact of the COVID-19 pandemic on paediatric health service use within one year after the first pandemic outbreak in New South Wales, Australia—a time series analysis. Lancet Reg Health West Pac. 2022;19:100311. doi: 10.1016/j.lanwpc.2021.100311 34746898 PMC8564784

[27] Williams TC, MacRae C, Swann OV, Haseeb H, Cunningham S, Davies P, et al. Indirect effects of the COVID-19 pandemic on paediatric healthcare use and severe disease: a retrospective national cohort study. Arch Dis Child. 2021;106(9):911–917. doi: 10.1136/archdischild-2020-321008 33451994 PMC8380881

[28] Bradshaw D, Dorrington R, Laubscher R, Groenewald P, Moultrie T. COVID-19 and all-cause mortality in South Africa—the hidden deaths in the first four waves. S. Afr. J. Sci. 2022;118(5/6). doi: 10.17159/sajs.2022/13300

[29] Sharkey AB, Chopra M, Jackson D, Winch PJ, Minkovitz CS. Pathways of care-seeking during fatal infant illnesses in under-resourced South African settings. Trans R Soc Trop Med Hyg. 2012;106(2):110–116. doi: 10.1016/j.trstmh.2011.10.008 22136954 PMC3254810

[30] Sayedy S. Impact of COVID-19 on the Severe Acute Malnutrition Admissions Among Children 1–5 Years of Age Seeking Nutrition Services in Afghanistan, 2021. (Masters Thesis, University of Arkansas) https://scholarworks.uark.edu/etd/4175.

[31] Summan A, Nandi A, Shet A, Laxminarayan R. The effect of the COVID-19 pandemic on routine childhood immunisation coverage and timeliness in India: retrospective analysis of the National Family Health Survey of 2019–2021 data. Lancet Reg Health Southeast Asia. 2023;8:100099. doi: 10.1016/j.lansea.2022.100099 36285007 PMC9584865

[32] Dalton M, Sanderson B, Robinson LJ, Homer CSE, Pomat W, Danchin M, et al. Impact of COVID-19 on routine childhood immunisations in low- and middle-income countries: A scoping review. PLOS Glob Public Health. 2023;3(8):e0002268. doi: 10.1371/journal.pgph.0002268 37611014 PMC10446229

[33] Bardsley M, Morbey RA, Hughes HE, Beck CR, Watson CH, Zhao H, et al. Epidemiology of respiratory syncytial virus in children younger than 5 years in England during the COVID-19 pandemic, measured by laboratory, clinical, and syndromic surveillance: a retrospective observational study. Lancet Infect Dis. 2023;23(1):56–66. doi: 10.1016/S1473-3099(22)00525-4 36063828 PMC9762748

[34] Parums Dinah. (2023). Editorial: Outbreaks of Post-Pandemic Childhood Pneumonia and the Re-Emergence of Endemic Respiratory Infections. Medical science monitor: international medical journal of experimental and clinical research. 29. e943312. doi: 10.12659/MSM.943312 38037346 PMC10702145

[35] Clarence E, Jeena P M. The unmet need for critical care at a quaternary paediatric intensive care unit in South Africa. SAMJ, S. Afr. Med. J. 112 (11): 871–878. doi: 10.7196/SAMJ.2022.v112i11.16452 36420729

